# A psychometric evaluation of the Multidimensional Social Competence Scale (MSCS) for young adults

**DOI:** 10.1371/journal.pone.0206800

**Published:** 2018-11-02

**Authors:** Dominic A. Trevisan, Donna Tafreshi, Kathleen L. Slaney, Jodi Yager, Grace Iarocci

**Affiliations:** 1 Faculty of Education, Simon Fraser University, Burnaby, BC, Canada; 2 Department of Psychology, Simon Fraser University, Burnaby, BC, Canada; 3 Compass Clinic, Vancouver, BC, Canada; University of Plymouth, UNITED KINGDOM

## Abstract

The current study contributes to previous work on measuring the social phenotype in Autism Spectrum Disorder (ASD) by validating a multidimensional test of social competence developed for use with individuals with and without ASD. The “Multidimensional Social Competence Scale” (MSCS) was previously validated as a parent-rating scale with youth 11–18 years with ASD without intellectual disability and typically developing adolescents of comparable age. The current study presents a validation of a self-report version of the MSCS in a non-clinical young adult population (*N* = 1178, *males* = 360, *females* = 817, *age range =* 17–25 years). The MSCS consists of seven domains that represent social competence: social motivation, social inferencing, demonstrating empathic concern, social knowledge, verbal conversation skills, nonverbal sending skills, and emotion regulation. These domains are theorized to be indicative of the higher-order construct of social competence. A second higher-order theorization of the MSCS structure posits that 3 of these factors are indicative of social responsiveness, and the remaining 4 factors are indicative of social understanding and emotion regulation. Our findings indicated support for each of the theorized multidimensional factor structures. Reliability, optimal scoring, convergent and discriminant validity of the measure, as well as implications for future research are discussed.

## Introduction

Autism Spectrum Disorder (ASD) is associated with a range of symptom severity and expression in children and adults [1]. Large individual differences in response to behavioural interventions imply etiological heterogeneity, and likely, heterogeneity at the level of underlying causal mechanisms [2–4]. “Person-oriented,” also referred to as dimensional, approaches have helped parse the heterogeneity observed in attention-deficit/hyperactivity disorder and antisocial behaviour [5–7]. The application of dimensional approaches represents a shift from the traditional emphasis on between-group differences to a focus on within-group differences [8–10]. Key to this approach is the identification of subgroups composed of individuals who are homogeneous regarding multiple variables considered simultaneously and throughout development [9]. Within a diverse population, there may be certain profiles that are particularly common and meaningfully categorized as subtypes. Such subgroups are not meant to represent “literally distinct groups” that map perfectly onto reality, but instead, are intended to “draw attention to differences in the causes and consequences” of their manifestations [11]. It is hypothesized that individuals within subgroups are more likely to share common etiological pathways and demonstrate similarities in treatment response and outcomes [12–13]. In contrast to categorical classifications systems (e.g., the Diagnostic and Statistical Manual of Mental Disorders[1]) that do not generally account for within-group heterogeneity, the person-centred or dimensional approach captures individual differences at the level of research data analysis as well as clinical assessment and treatment.

One approach to identifying homogeneous subgroups involves identifying common profiles with respect to a single domain of interest. By affording a greater level of specificity, this approach would allow researchers to ask more targeted questions of their data [12]. In the current research, we focus on the domain of social competence. We aim to contribute to previous work on assessing the social phenotype in ASD by validating a multidimensional test of social competence for use with adults with and without ASD.

### The social phenotype in ASD

Given that the social domain may be the most defining area of impairment in ASD [13–14], the identification of homogeneous subgroups based on social functioning may shed light on meaningful sources of heterogeneity within ASD. Although all individuals with ASD meet the diagnostic criteria for social impairment, there is a large range of expression and severity of social deficits apparent within this population. Wing and Gould described three different categories of social impairment that might explain some of this variability: “aloof,” “passive,” and “active but odd” [15]. They described individuals in the first classification, “aloof,” as being socially withdrawn, while individuals in the second category, “passive,” as being open to social contact but unlikely to initiate it. The third classification, “active but odd,” describes individuals who initiate social interactions in an inappropriate manner. These categories appear to be differentiated primarily by varying levels of social motivation–ranging from a lack of interest (aloofness) to passive acceptance to heightened social interest but odd approach behaviour. Variability in social interest is a potentially discriminating factor as some individuals with ASD may be more likely to withdraw or show disinterest in others while others may desire social interaction but demonstrate their interest in inappropriate or awkward ways [16–18].

Yager and Iarocci developed and validated the “Multidimensional Social Competence Scale” (MSCS) as a parent-rating scale with youth aged 11–18 with Autism Spectrum Disorder (ASD) without intellectual disability and typically developing (TD) youth of comparable age [19]. This is because the social difficulties associated with ASD may in fact intersect with the variability seen in social competence within the general population [4]. As such, the MSCS was designed to assess a range of social behaviours frequently observed in high-functioning ASD individuals and that might also be seen in TD individuals exhibiting social impairment to a lesser degree.

As a next step toward the goal of developing a general measure of social competence, we extend the utility of the MSCS for use as a self-report scale in the general population and with older individuals, particularly young adults (ages 17–25). Given that this age range reflects the transition from adolescence into adulthood, variability observed in social competence should be particularly salient during this time. Thus, adapting procedures outlined by Slaney and Maraun [20], we conducted a test validation of a self-report version of the MSCS to assess whether its theorized multidimensional structure holds within a non-clinical young adult population.

### Measures of social competence

Although several valid and widely used measures of social competence are available, these tests were designed for assessing specific aspects of social functioning in particular populations. For example, the “Social Skills Rating System” (SSRS) assesses social skills, problem behaviours, and academic competence [21]. The SSRS is useful for school assessments aimed at identifying individuals at risk for academic and behavioural problems. The social skills domain focuses primarily on broad-based social competencies specific to school settings (e.g., cooperation, responsibility and self-control) but is not likely to capture the finer-grained skills (e.g., social inferencing and verbal and nonverbal communication skills). Moreover, the scale is restricted to use with school-aged individuals.

Measures such as the “Autism Spectrum Quotient” (AQ), the “Broad Autism Phenotype Questionnaire” (BAPQ), and the “Social Responsiveness Scale” (SRS) were designed to assess the presence and extent of autistic traits [22–24]. Given that ASD is often conceptualized as a social disorder, measures of autistic traits such as the AQ are often used as measures of social ability. However, several clinical features and characteristics of ASD are non-social in nature—therefore, in addition to social items, the SRS measures repetitive behaviours/interests [25] and the AQ measures unusual and restricted interests, cognitive thinking styles, and executive functioning. Although these ASD screening tools are useful to screen for ASD symptoms and traits in the population at large, these measures do not extend well to measuring social competence in the general population because they include the assessment of features of autism that are not social in nature, such as, sensitivity to sensory stimuli and repetitive interests. This introduces construct-irrelevant variance when these measures are used to measure social competence with nonclinical populations. The MSCS differs in that it was designed based on a broad literature on social ability and development rather than an exclusive focus on social impairment related to symptoms of ASD.

Also widely used to assess social competence is the standardized “Vineland Adaptive Behaviour Scales” (VABS) [26–27]. The VABS measures social and non-socially adaptive and maladaptive behaviours. Although it is appropriate for use with all ages, the VABS was specifically designed to assess adaptive behaviour. According to the authors “the scales are organized using three domains—Communication, Daily Living Skills, and Socialization—that correspond to the three broad domains of adaptive functioning specified by the American Association on Intellectual and Developmental Disabilities and by DSM-5” [1,28]. The Socialization domain measures subdomains of Interpersonal relationships, Play and Leisure Skills and Coping Skills. The items on this subdomain and, more broadly on the VABS, focus on functional behaviours “Goes places with friends …”; “plans fun activities…” The MSCS has a somewhat different focus on social behaviours that reflect competencies in social situations. For example, “I recognize when people are trying to take advantage of me” or “I dominate conversations so that it can be hard to get a word in”. Thus the MSCS may better capture the heterogeneity in finer-grained social competencies in the general population, or in individuals with ASD with average and above average intelligence.

The “Emotional Quotient Inventory” (EQ-i) is a test of emotional-social intelligence that was originally designed for adults [29] but has since been adapted for use in child and adolescent populations [30]). This inventory measures some aspects of social and emotional competencies such as the abilities to identify and regulate emotions in others, relationship success, social awareness, stress management, adaptability, and general mood. The EQ-i is often administered in vocational settings used to make hiring decisions based on Bar-On’s[29] assertion that emotional intelligence contributes to real-world educational and workplace success. However, the EQ-i has been criticized for being based on a vague theoretical background that has not held up to empirical scrutiny [31]. For example, while emotional intelligence is conceptualized as a facet of intelligence, the EQ-i appears to be more strongly related to personality and psychopathology than related to intelligence, cognitive abilities or predictive of real-world success [30, 32–33] The MSCS includes emotion regulation as one dimension of social competence but also has 6 other dimensions that are theoretically and empirically related to the construct of social competence.

### Development of the MSCS

According to the prism model, social competence can be measured at two overarching levels [19, 34]. The *index* level measures real-life indices of social competence, such as relationship quality, peer acceptance, and employment success. These indices do not provide detailed information about the social presentation of an individual but rather, are useful outcome measures. The *motivation/skills* level comprises the specific aptitudes demonstrated during social interactions including social-cognitive abilities and overt behaviours (e.g. eye contact). The MSCS was designed to parse the heterogeneity in social competence by identifying *motivation/skills* level behaviours and abilities that underlie successful social interactions at [19, 34]. Such indices have, for the most part, been overlooked in existing measures [35].

### Originality of the MSCS

The MSCS is novel in that it recognizes that social competence exists in dynamic interactions between individuals and is multidimensional in nature. For example, the MSCS examines key transactional and relational aspects of social interactions (e.g., conversational turn taking, entering and leaving conversations, social referencing) in detail [36–37] and its items include individual and interpersonal behaviour [38], skillful coordination of multiple cognitive processes and the integration of contextual factors (e.g., social norms) to adequately reflect the social demands of different situations. We theorize that social competence 1) changes over the course of development, 2) emerges within a dynamic interaction between individuals and 3) is multidimensional in nature. However, we cannot capture all of these aspects of social competence in the MSCS. Thus, we developed the MSCS to primarily tackle the multidimensional aspect of SC; differentiating social motivation from social understanding and identifying 7 relatively distinct subdomains. The main goal is to identify more homogenous groups of participants for research studies or clinical interventions based on social competence profiles. We suspect that any item on the MSCS (e.g., demonstrating empathic concern) would have consistency but also differences across settings. However, the MSCS is mostly tapping consistency across contexts. That is, the individual would be rating how they feel they demonstrate their empathic concern across many contexts. For clinical purposes, the clinician on our team (GI) has found it helpful to further discuss an item where there is an uncertain response (e.g., “it depends”) and gather more specific information about the factors that contribute to differences across contexts (e.g., sensory overload) as this may be especially helpful in the design of therapeutic strategies. However, the MSCS is not yet fully developed for clinical use and thus, does not yet have this feature that allows one to qualify a response, however, it is something that is being considered in refining its use for clinical purposes.

### Utility of the MSCS

The MSCS fills a gap in the current repertoire of social measures; it captures aspects of social functioning that are fine-grained, multi-dimensional, and not disorder-specific. Therefore, the MSCS will more precisely compare levels of social functioning among different populations and be especially useful for discerning shared and distinct patterns of social competence across different populations characterized by social impairment [39]. Direct comparisons across clinical populations can reveal disorder-specific aspects of social impairment missed when using traditional approaches comparing clinical groups to typically developing comparison groups [40]. The MSCS may also be useful alongside diagnostic tools such as the Autism Diagnostic Observation Scale (ADOS) and the Autism Diagnostic Inventory-Revised (ADI-R) for the purposes of developing quantitative profiles, parsing heterogeneity in social competencies and subtyping individuals with ASD [41–42].

### Theoretical structure of the MSCS

Although not exhaustive, the motivations/skills selected for the MSCS were intended to be representative of the variables associated with peer acceptance and close friendship development. The MSCS consists of 7 content domains [19]. First, *social motivation* reflects an individual’s desire to interact with others [35]. This domain measures, to some degree, the *reward value* individuals obtain from social interactions. Accordingly, items on this domain measure the degree to which respondents initiate social contact, have apparent interest in friendships and other relationships, desire to be accepted by peers, and derive enjoyment from social interactions.

*Social inferencing* represents the capacity to detect social cues, especially regarding making accurate judgments of others’ mental states and reading social situations in a way that guides subsequent appropriate behaviour. These skills are important considering that people’s mental states may not always match their verbal expressions.

*Empathic concern* represents the ability to respond to others’ emotional states in a sensitive way. Empathic responses can occur through sharing the feelings of others or feeling compassion in response to someone’s distress [43]. Empathic concern is related to social inferencing. That is, to react to others’ emotional states with an appropriate empathic response, one must first be able to correctly *interpret* others’ emotional states [44].

*Social knowledge* refers to the ability to understand the “procedural rules” of social situations and to modify one’s social behaviour in response to the demands and features of such situations. Such knowledge also reflects understanding of how one might engage differently with peers as opposed to authority figures, as well as the ability to identify potentially risky or dangerous social situations.

*Verbal conversation skills* refer to the ability to engage in reciprocal conversation. Attributes in this domain reflect the ability to take turns, to understand how to initiate, join and leave conversations, and to know when to maintain or change conversational topics.

*Nonverbal sending skills* reflect the ability to use body language to facilitate communication (e.g., eye contact, facial expressions, gestures). This domain also reflects competencies such as knowing when to smile, looking concerned in response to another’s distress, as well as nonverbal aspects of speech related to prosody, style, and volume.

Finally, *emotion regulation* refers to the processes involved in monitoring, evaluating, and controlling the intensity of one’s internal emotional experiences and outward emotion-related behaviour to attain desired affective states. Table 1 lists all 77 of the MSCS items organized according to content domain. The scale is available in a usable format in the supplementary material for readers to download to use for their own research or clinical purposes.

**Table 1:** MSCS items organized by domain.

**Social Motivation**
*1. I prefer to spend time alone (e.g., I am most content when left on my own).
*42. I need to be told or prompted to talk or interact with people.
*14. I stay in the “background” in group social situations (e.g., keep to myself, may not be noticed).
*69. I show little interest in people.
*22. I avoid talking to people when possible (e.g., look, move, or walk away).
**2.** I enjoy meeting new people.
**76.** I introduce myself to people (without being told to).
**10.** I initiate friendly social “chit-chat” with people (e.g., ask about what’s new with other person, talk about the weather or events). These are casual conversations that often have no specific purpose.
**57.** I seek out people to spend time with (e.g., friends, other people).
**65.** I initiate get-togethers with peers (e.g., call or email or text them to make plans).
**19.** I ask people questions about themselves or their lives (e.g., how they are, what they’ve been up to).
**Social Inferencing**
**54.** I recognize when people are trying to take advantage of me.
**77.** I understand when people are being sarcastic.
*52. I have trouble judging who is trustworthy (e.g., who to share secrets or personal information with).
*13. I misread social cues.
*67. I do not pick up on the subtleties of social interaction.
**45.** I can see things from another person’s perspective.
**3.** I easily recognize unfriendly actions. For example, I know when someone is making fun of me in a mean-spirited way. Or, I recognize when a peer is pressuring me to do something I shouldn’t or don't want to do.
**28.** I pick up on subtle hints and indirect requests. For example, I would understand that when someone asks “Can you reach that book?”, they are asking me to pass it to them. In other words, I can “read between the lines” when others are talking.
*40. I am naïve (believe whatever I am told).
*59. I have trouble predicting what other people will do or how they will react.
**24.** I can tell when people are joking.
**Demonstrating Empathic Concern**
**9.** I am sensitive to the feelings and concerns of others.
**16.** I express concern for others when they are upset or distressed (e.g., may ask “are you alright?” or ask if they need anything).
**48.** I offer comfort to people (e.g., to someone who is upset, not feeling well, hurt etc.). For instance, I may try to hug the person or provide a comforting object as a way of trying to make the other person feel better.
**64.** I congratulate people when good things happen to them.
**55.** I try to cheer people up (when they are down).
**5.** I apologize after hurting someone (without being prompted or told to).
*27. I am indifferent or “oblivious” to people who are upset (or in distress).
**39.** I am concerned about people and their problems (e.g., talk to someone who is having a hard time).
**30.** I appear visibly upset when I see people suffering (in real life or on tv/film).
*20. I give compliments to people.
**11.** I do not offer to help people (unless asked or told to).
**Social Knowledge**
**26.** I know about the latest trends for my age (e.g., in clothes, music, tv shows/movies, music).
**72.** I change my behaviour to suit the situation. For example, I might be more polite/ formal around authority figures like teachers or supervisors but be more casual around peers. As another example, I might change my way of speaking depending on who you are talking to (e.g., talk more simply to a younger child).
**73.** I dress appropriately for my age and social situation (e.g., dress up for formal events, wear more casual clothes on weekends, wear clothes that are generally considered acceptable by peers my age).
**33.** I change the volume of my voice depending on where I am (e.g., quiet at the library, movies but louder when outside or at a sporting event).
**75.** I hide my true feelings (when necessary) so that I don’t come across as rude (e.g., I might hide feelings of disappointment when given a gift that I do not like or when someone breaks something of mine by accident).
**58.** I understand the “social hierarchy” at school or work or in other settings (e.g., understand that teachers or supervisors are in a position of authority).
**43.** I follow social “rules” around privacy (e.g., respect people’s privacy when they are changing/ in the washroom; knock on closed doors instead of barging in).
**71.** I understand that it is important to have good personal hygiene (e.g., smelling and looking clean).
**53.** I understand what makes a true friend.
**31.** I act appropriately for my age in public (e.g., restaurants, movie theatres, libraries, doctor’s waiting rooms, etc).
**47.** My expectations of friends reasonable. For example, I know that they have other friends or are not always available.
**Verbal Conversation Skills**
*61. I dominate conversations so that it can be hard for others to “get a word in”. For example, I might ramble on and on about a favourite topic of interest. I might also need reminders/prompting to let others speak.
*7. I shift conversations to my favourite topic or interest.
*8. I talk about the same things over and over (“get stuck” on certain topics).
*63. I provide too much detail when talking about a topic (e.g., I might list a bunch of facts rather than expressing a main message or exchanging information).
*6. I talk “over” people in conversations (e.g., interrupt a lot, don’t wait for others to finish speaking).
*74. I talk too much.
**56.** I give other people a chance to speak during conversations (e.g., pauses, asks them questions).
*12. I have trouble joining conversations appropriately (e.g., I may interrupt or “butt in” without waiting for a good time to join in; or, I may start talking about a topic of interest to me regardless of the ongoing conversation).
*37. I talk “at” people (e.g., almost like I am giving a lecture).
*38. I go off track during conversations (e.g., I might change topics suddenly as if thinking aloud or reminded of something else; or, I might gradually get sidetracked or lose track of your original point).
**50.** I am good at taking turns in conversations (e.g., my conversations have age -appropriate levels of back-and-forth with each person getting a chance to talk; I respond appropriately to the other person’s questions or statements).
**Nonverbal Conversation Skills**
*51. My facial expressions seem “flat” (e.g., my face may be like a “blank slate” or seem overly serious).
*62. I sound the same (have the same tone and intonation in his/her voice) regardless of how I am feeling. In other words, it is hard to tell what I am feeling based on the way my voice sounds.
**23.** My facial expressions are easy to read.
**17.** I look people in the eye when talking to them.
*29. My smiles seem forced or awkward.
**66.** I point at things when appropriate (e.g., to get another person to look at something far away).
**49.** I use appropriate gestures when communicating with people (e.g., nodding/shaking head, waving goodbye, pointing at something interesting or far away, giving thumbs up, putting finger to lips for “be quiet”, etc.).
**34.** I show a range of facial expressions (e.g., embarrassed, guilty, surprised, disgusted, pleased).
*70. I speak with a flat, monotonous tone of voice.
**35.** I smile appropriately in social situations (e.g., if given a compliment, greeting someone, in response to someone smiling at me).
**32.** I use eye contact to get other people’s attention (e.g., to start a conversation, ask a question).
**Emotional Regulation**
*46. I have “meltdowns” (e.g., sudden outbursts, “blow ups” temper tantrums).
*18. I get frustrated easily.
**15.** I am patient (e.g., when waiting).
*36. I act out when angry or upset (e.g., yell at, hit, or shove people).
*21. My emotional responses tend to be extreme (e.g., I might be extremely angry or frustrated in response to relatively small problems).
*60. I get very upset if things are not done your way.
**41.** I get over setbacks or disappointments quickly.
*44. I get very anxious.
**4.** I disagree with people without fighting or arguing.
*68. My emotions tend to be “all or nothing” (“all on” or “all off”).
**25.** I stay calm when problems come up.

*Items marked with an asterisk are reversely keyed.

In addition to the conceptualization of the theoretical structure of the MSCS as consisting of seven domains that feed into the higher-order construct of social competence, Yager and Iarocci [19] conducted exploratory factor analyses that suggested a possible second, higher level, structure of the MSCS. Based on factor correlations, they theorized that the domains of social motivation, empathic concern, and nonverbal skills assessed the “social responsiveness” aspect of social competence; whereas, the social inferencing, social knowledge, verbal skills, and emotion regulation domains related to the “social understanding/emotion regulation” aspects of social competence. These models are different but not mutually exclusive. The objective of the current analysis was not to find the “right” model (as many models may provide a similar fit to a given dataset; see Slaney and Maraun, 2008 [20]), but to determine whether both general and fine-grained conceptualizations of social competence are reflected in the seven domains of the MSCS. The goals were, first, to determine whether the two theoretical structures hypothesized by Yager and Iarocci [19] would be replicated for a self-report version of the MSCS and a population of young adults and, second, to demonstrate whether scoring is justified in the current sample at the domain, subscale, and total scale levels.

### Self vs. parent report versions of the MSCS

The MSCS was originally developed as a parent-report measure and was adapted as a self-report measure for the current study. Self-report measures have generally been overlooked in research on individuals with ASD because of the concern that they may have difficulty engaging in introspection [45–46]. However, in the case of individuals with ASD with average intellectual functioning (IQ > 85), there is evidence to suggest agreement between youth and their parents in reporting social and psychiatric symptoms [47–48] as well as adequate validity indexes on broad base behavioural measures for youth with ASD [49]. The subjective viewpoint of experience provided by self-report measures is not available with other methods of assessment and offers unique insight on social competence.

The theoretical foundations of the MSCS remain the same for the adolescents in the previous parent-report study and young adults in the current study. Thus, the same theorized structures of the MSCS for the self- and parent-report versions of the test are hypothesized in the present study. On these grounds, we explored the psychometric properties of the test using confirmatory, rather than exploratory, factor analysis.

### Current study

The objectives of the present study were twofold. First, we aimed to examine whether the theoretical structure of the MSCS identified by Yager and Iarocci [19] would hold for self-ratings on the MSCS in a non-clinical sample of young adults. To this end, we did preliminary testing of the unidimensional measurement models for each of the 7 domains of the MSCS before testing the multidimensional structure hypothesized by each of two structural models: a) a 7 first-order (i.e., 7 domains) and 1 second-order (i.e., social competence) hierarchical linear factor model; and b) a 7 first-order (i.e., 7 domains) and 2 second-order (i.e., social responsiveness and social understanding/emotion regulation) hierarchical linear factor model. Our second aim was to determine on the basis of our model fit tests which scoring procedures were justified for this sample to assess the external validity of the MSCS. Convergent validity of the MSCS was examined through correlations of MSCS model-derived weighted domain scores with theoretically related measures.

## Method

### Participants

Data was collected from 12 studies involving typically developing Canadian undergraduate university students (*N* = 1391). All participants provided written consent to participate in this study. In Canada, individuals aged 16 or older can provide written consent to participate in research without the consent of a guardian. This study received ethics approval from our Simon Fraser University’s Research Ethics Board (# 2014s0694). Participants who fell within the age range of 17.5–25.5 years were included in the analyses. Participants were excluded (N = 112) because they fell outside the specified age range or their age was missing. Three participants were missing information pertaining to one of the MSCS items. These missing items were imputed using mean substitution based on the average score for that person’s valid test items. Thirty-three participants had 5 or more missing items on the MSCS and were excluded from the analyses. Sixty-eight individuals participated in more than one of the 12 studies; in these instances, only the participant’s first testing session was included in the analyses. The final sample of 1178 young adults consisted of 360 males and 817 females. Ages ranged from 17 years, 6 months to 25 years, 6 months (*M* = 19.72, *SD* = 1.61).

### Materials

#### Multidimensional Social Competence Scale (MSCS)

Of the sample of 1178, 1038 participants completed an online version of the self-report MSCS (administered using a university WebSurvey software program) and 140 participants completed the paper version. Both versions had identical questions and contained a total of 77 items, with each of the 7 content domains containing 11 items. Participants were asked to rate each of these items on a 5-point Likert scale, with higher scores indicating greater agreement with an item, and thus, higher levels of social competence.

#### Autism Spectrum Quotient (AQ)

The AQ [22] is a self-report questionnaire used to screen for social and non-social autism symptomology. As the AQ is commonly used to assess social competencies, the full scale AQ was correlated with the full scale MSCS to assess convergent validity. Additionally, the AQ has two subscales that are specifically related to social competencies: “social skills” and “communication skills.” These subscales were used to assess convergent validity of the MSCS social knowledge and MSCS verbal conversation skills domains, respectively. Two subscales of the AQ, “Attention to Detail” (tendency to notice small details and patterns, rather than focusing on the ‘big picture’) and “Attention Switching” (tendency to prefer ritualized routines and hyper-focused attention to special interests) which are less theoretically related to social competence, were used to demonstrate discriminant validity of these same MSCS domains. As higher scores on the MSCS represent higher social *competency*, whereas higher scores on the AQ represent social *impairment*, the observed correlations were expected to be negative.

#### Behaviour Rating Inventory of Executive Function (BRIEF)

The BRIEF assesses executive dysfunction related to behavioural regulation and metacognitive skills [50]. The “monitor” subscale of the BRIEF assesses impairment in the ability to monitor the effect of one’s own behaviour on others. We reasoned that accuracy in assessing one’s influence on others would require the ability to make accurate social inferences. As such, the monitor subscale of the BRIEF was used to measure convergent validity of MSCS social inferencing domain scores. The BRIEF “emotional control” subscale was used to assess convergent validity of MSCS emotion regulation domain scores given the similarity of content between these subscales. To demonstrate discriminant validity of these two MSCS domains, we used two subscales of the BRIEF, “Initiate” (ability to begin activities and independently generate ideas and solutions), and “Organization of Raw Materials” (ability to organize one’s work space and personal belongings), both of which are less theoretically relevant to social competence. We also used a subscale of the BRIEF, “Working memory” (ability to hold information when completing a task or encoding information), to demonstrate discriminant validity of “Empathic Concern.” Observed correlations were expected to be negative as higher social competence was expected to be associated with lower executive dysfunction.

#### Behaviour Assessment System for Children (BASC-2) self-report-college

The BASC-2 assesses adaptive and maladaptive behaviours of college students [51]. We reasoned that higher scores on the BASC-2 “social stress” subscale would be negatively correlated with behaviours indicative of initiating, seeking, and enjoying social interactions as measured by the MSCS “social motivation” domain. We used a subscale of the BASC, “Inattention/Hyperactivity”(which measures traits associated with Attention Deficit and Hyperactivity Disorder) to demonstrate discriminant validity for the social motivation domain. We also used the “Anxiety” subscale of the BASC to demonstrate discriminant validity for “Nonverbal sending skills” and used the “Somatization” (complaints of physical symptoms that have no medical basis) subscale of the BASC to demonstrate discriminant validity for the full scale MSCS. Again, these subscales of the BASC were used for discriminant validity based on their minimal theoretical overlap with social competence.

### Diagnostics

Prior to combining data from the different studies, we ran one-way analyses of variance (ANOVAs) to address any systematic differences between participants’ MSCS domain scores based on the study in which the participant was tested. For the domains of social motivation, social inferencing, empathic concern, verbal skills, and nonverbal skills, the omnibus *F*-test did not indicate any statistically significant differences in non-weighted composites between the 12 different studies at a pre-set alpha level of .05 (*F*’s (11, 1166) ranged from .666 to 1.324). However, the omnibus *F* statistics for the social knowledge and emotion regulation domains were statistically significant (*F’*s (11, 1166) for social knowledge = 2.343 and for emotional regulation = 2.737). Further, Tukey post-hoc comparisons showed that these differences were mainly attributed to 2 studies. Thus, we ran the subsequent CFA analyses both including and excluding these 2 studies. We observed only slight differences in fit indices and factor loadings. There is no theoretical reason for us to believe that these 2 samples might have characteristically differed from the rest of the samples in the study, therefore, we opted to report the results including all 12 samples.

We computed bivariate correlations between each of the item scores within the 7 domains to confirm that items within the same domain were positively correlated with one another as theorized. The results revealed that item 41 of the emotion regulation domain was negatively correlated with almost all other items in that domain. It may be the case that item 41, “I get over setbacks or disappointments quickly”, behaved differently due to the self-report nature of the MSCS used in the present study. Yager and Iarocci’s [19] results, which were based on a parent-report version of the test, indicated a positive factor loading for this item. One possibility is that this item is indicative of emotion regulation only when parents score their children, but not when a young adult self-reports. We recommend researchers using the MSCS examine bivariate correlations between item 41 and other domain items; if correlations are negative, the emotion regulation domain can be assessed with the omission of item 41. Accordingly, we chose to omit item 41 from the current analyses.

### Data analysis plan

**Fig 1:**
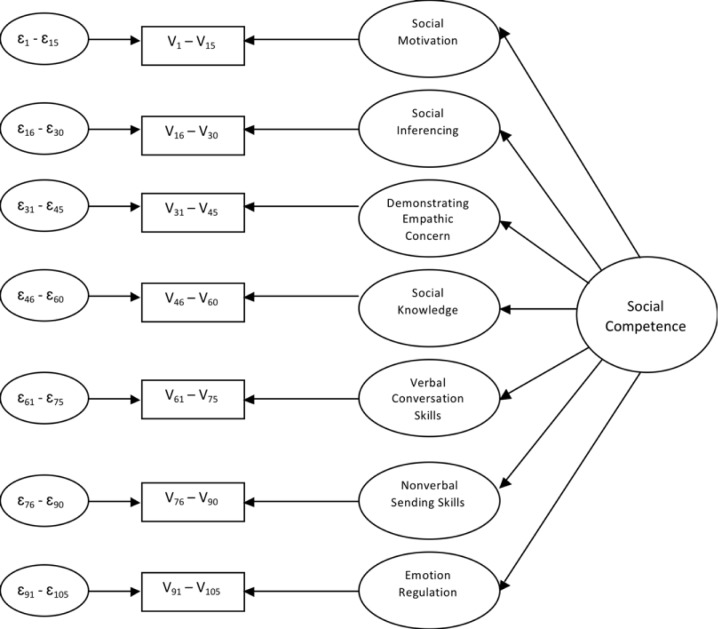
Model 1: 7 first-order factors and 1 second-order factor.

First, we tested the measurement model for each domain separately with 1-factor linear models using confirmatory factor analysis (CFA). For each model, we constrained the item responses for the 7 domains (each consisting of 11 items, apart from the emotion regulation domain which consisted of 10) to load onto a single factor; loadings and error variances were left to vary (i.e., were specified as 1-factor “congeneric” models [52]). Next, also using CFA models, we examined the two hypothesized multidimensional theoretical structures of the MSCS proposed by Yager and Iarocci [19]. The first model tested a 7 first-order factors (representing the 7 domains of the MSCS) and 1 second-order factor (social competence) structure (see Fig 1). Here, factor loadings could vary, whereas, the variance of the higher-order factor (“social competence”) and the error variances for each of 7 first-order factors were fixed at 1.

**Fig 2:**
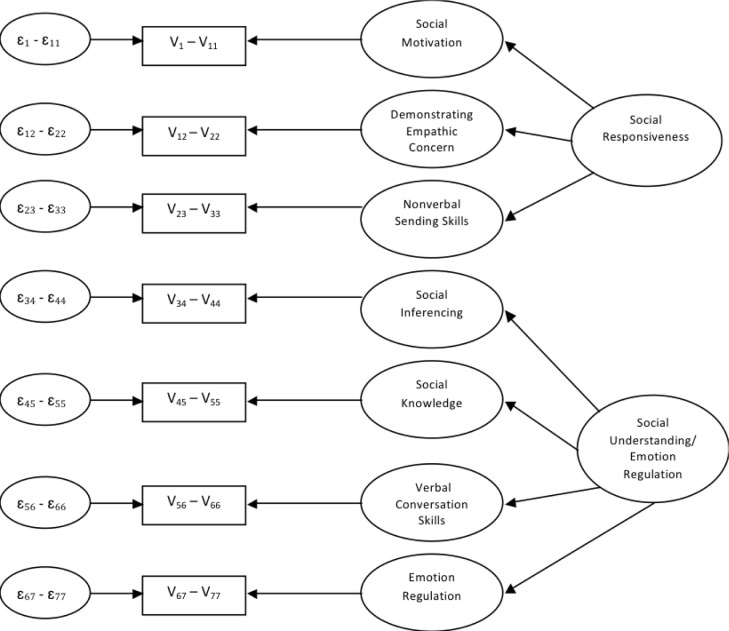
Model 1: 7 first-order factors and 2 second-order factors.

The final CFA tested a linear model with 7 first-order factors and 2 second-order factors identified in Yager and Iarocci [19] (see Fig 2). In that study, the first higher-order factor was labeled “social responsiveness” and the second higher-order factor was labeled “social understanding/emotion regulation”. Thus, we constrained domain scores for social motivation, empathic concern, and nonverbal skills to load on the first higher-order factor, and the domain scores from the remaining 4 domains to load on the second higher-order factor. As with the first multidimensional model, each of the factor loadings could vary, whereas, the variances of the 2 higher-order factors and the error variances of each of the 7 first-order factors were fixed at 1.

Next, on the basis of our model fit tests, we created weighted composites for each of the domain scores, as well as for the overall MSCS, social responsiveness, and social understanding/emotion regulation total scores. Weights were created by dividing the factor loadings by their corresponding error variances for each item across the different models. We used omega-squared to estimate reliability of the weighted composites across the 3 models and coefficient alpha to estimate the reliability of non-weighted composites.

Finally, given that data reported in this article were extracted from a larger database, convergent measures were only available for portions of the entire sample and for some of the MSCS domains. In total, we identified relevant measures for the full scale MSCS and 5 of the 7 MSCS domains. We ran correlational analyses to assess the convergent validity of each of these composites. All correlation estimates were corrected for attenuation.

## Results

### Confirmatory factor analyses

CFAs were conducted using the CALIS procedure in SAS Software 14.0 on the sample covariance matrix using diagonally weighted least squares estimation (based on the ordinal nature of the test data). We used two measures of absolute fit: the standardized root mean square residual (SRMR) and the root mean square error of approximation (RMSEA). Moschopoulos’ [53] approximation was used for computing the 90% confidence intervals around the RMSEA estimates. We employed Hu and Bentler’s [54] criteria for “relatively good fit” for a sample size N>250. SRMR values less than .08 are ideal, and RMSEA values less than .06 are ideal, although, RMSEA values between .06 and .08 are often considered acceptable. We also report the chi-square (*χ*^2^) estimate; however, given that chi-square is a biased measure of fit when N>200, we emphasize the SRMR and RMSEA estimates [55]. In addition, readers should note that Hu and Bentler’s [54] guidelines are specific to maximum likelihood estimation. However, Zhao [56] examined the use of fit indices and Hu and Bentler’s [48] guidelines with diagonally weighted least squares estimation. He recommended that if the *χ*^**2**^ statistic is small and the RMSEA value is small, then one can conclude adequate model fit. Alternatively, if the *χ*^**2**^ statistic is large and the RMSEA value is small, but the sample size is very large, then researchers might conclude that the model fits adequately because the inflated *χ*^2^ value is due to the large sample size.

#### Domain measurement models

Table 2 presents the results of 1-factor CFAs. The SRMR and RMSEA indices were adequate for all dimensions. This indicates support for each of the domain measurement models and the forming of unweighted composites (i.e., simple sums of items.

**Table 2:** Indices of fit for domain measurement models.

Factor	*χ*^2^ (df)	SRMR	RMSEA	RMSEA 90% Lower CL	RMSEA 90% Upper CL
Social Motivation	182.435(44)	.0537 ^a^	.052 ^a^	.045	.058
Social Inferencing	145.477(44)	.048 ^a^	.044 ^a^	.038	.050
Demonstrating Empathic Concern	82.460 (44)	.037 ^a^	.027 ^a^	.021	.033
Social Knowledge	120.996(44)	.077 ^a^	.039 ^a^	.033	.044
Verbal Conversation Skills	240.226(44)	.061 ^a^	.062 ^a^	.055	.068
Nonverbal Conversation Skills	382.760 (44)	.064 ^a^	.081 ^a^	.073	.088
Emotion Regulation	94.984 (35)	.042 ^a^	.038 ^a^	.032	.044

^a^ Acceptable fit index.

#### Theoretical structural model 1: 7 first-order factors and 1 second-order factor

The SRMR and RMSEA estimates for Model 1 indicate adequate fit (see Tables 3 and 4). The estimated loadings for each of the domains on the second-order factor were positive. Table 5 presents the estimated correlations for the model.

**Table 3:** Indices of Fit for multidimensional structural models.

Model	*χ*^2^ (df)	SRMR	RMSEA	RMSEA 90% Lower CL	RMSEA 90% Upper CL
7 First-Order & 1 Second-Order Factors	15898.563(2772)	.073 ^a^	.063 ^a^	.057	.070
7 First-Order & 2 Second-Order Factors	14301.727(2768)	.070 ^a^	.059^a^	.053	.066

^a^ Acceptable fit index.

**Table 4:** Standardized factor loadings for first–order factors.

Model	Social Motivation ^b^	Social Inferencing ^a^	Empathic Concern ^b^	Social Knowledge ^a^	Verbal Skills ^a^	Nonverbal Skills ^b^	Emotion Regulation ^a^
7 First-Order & 1 Second-Order Factors	.723	.723	.767	.766	.804	.804	.470
7 First-Order & 2 Second-Order Factors	1.183	1.455	1.308	1.909	4.042	2.731	.615

^a^ First-order factors loading onto the “social understanding/emotion regulation” second-order factor in Model 2.

^b^ First-order factors loading onto the “social responsiveness” second-order factor in Model 2.

**Table 5:** Estimated correlations between 7 composite domain scores and MSCS total score.

	Social Motivation	Social Inferencing	Empathic Concern	Social Knowledge	Verbal Skills	Nonverbal Skills	MSCS Total Score
**Social Motivation**	—	—	—	—	—	—	.712
**Social Inferencing**	.381	—	—	—	—	—	.702
**Empathic Concern**	.542	.306	*—*	*—*	*—*	*—*	.683
**Social Knowledge**	.352	.541	.458	—	—	*—*	.699
**Verbal Skills**	.112	.373	.212	.382	—	*—*	.584
**Nonverbal Skills**	.563	.420	.568	.485	.284	—	.758
**Emotion Regulation**	.269	.378	.143	.265	.467	.758	.599

#### Model 2: 7 first-order factors and 2 second-order factors

The fit indices for Model 2 were slightly better than that of the second model, again indicating reasonably good fit (see Table 3). The estimate of the covariance between the 2 second-order factors was .731. Tables 3 and 4 present the factor loadings for the domains on the two second-order factors.

### Reliability and convergent validity

The results of the structural model tests indicate that there is justification for forming composite scores for the 7 MSCS domains, the overall MSCS, as well as the social responsiveness and social understanding/emotion regulation subscales. Given that the measurement models for all 7 domains indicated adequate fit, forming unweighted item composites for each of the 7 domains and interpreting these as indicators of unidimensional is supported for this data. Reliability estimates (i.e., Chronbach’s alpha) for the unweighted composites of each of the domains and higher order composites are presented in Table 6. Table 6 also presents the results of correlation analyses assessing convergent validity, and Table 7 presents the results of correlation analyses assessing discriminant validity. Domain scores and total scores correlated with each of the external measures in the expected directions.

**Table 6:** Convergent validity coefficients and internal consistency reliability of measures.

MSCS Measure	α	Convergent Measure	α	Observed *r*	*P*	*N*	Non-Attenuated *r*
Emotion Regulation	.826	BRIEF–Emotional Control	.894	-.753	< .001	329	-.877
Verbal Conversation Skills	.794	AQ–Communication Skills	.593	-.437	< .001	838	-.687
Social Knowledge	.739	AQ–Social Skills	.677	-.322	< .001	838	.503
Social Inferencing	.793	BRIEF–Self Monitor	.700	-.353	< .001	329	.513
Social Motivation	.862	BASC-2 –Social Stress	.732	-.548	< .001	330	.726
Nonverbal Sending Skills	.806	*None Available*	—	—	—	—	—
Empathic Concern	.839	*None Available*	—	—	—	—	—
Full Scale	.795	AQ–Full Scale	.766	-.662	< .001	838	-.775
Social Responsiveness Total Score	.784	*—*	—	—	—	—	—
Social Understanding/Emotion Regulation Total Score	.720	*—*	—	—	—	—	—

*Note*. Item #41 removed for MSCS-Full Scale and MSCS-Emotion Regulation.

**Table 7:** Discriminant validity coefficients and internal consistency reliability of measures.

MSCS Measure	α	Discriminant Measure	α	Observed *r*	*P*	*N*	Non-Attenuated *r*
Emotion Regulation	.826	BRIEF–Initiate	.750	-.248	< .001	329	-.315
Verbal Conversation Skills	.794	AQ–Attention to Detail	.597	-.040	.245	838	-.058
Social Knowledge	.739	AQ–Attention Switching	.514	-.185	< .001	838	-.300
Social Inferencing	.793	BRIEF–Organization of Raw Materials	.858	-.099	= .074	329	-.120
Social Motivation	.862	BASC-2 –Inattention/ Hyperactivity	.704	-.194	< .001	330	-.249
Nonverbal Sending Skills	.806	BASC—Anxiety	.773	-.142	= .010	330	-.180
Empathic Concern	.839	BRIEF–Working Memory	.800	.022	= .691	329	.027
Full Scale	.795	BASC–Somatization	.712	-.151	= .006	330	-.201
Social Responsiveness Total Score	.784	*—*	—	—	—	—	—
Social Understanding/Emotion Regulation Total Score	.720	*—*	—	—	—	—	—

*Note*. Item #41 removed for MSCS-Full Scale and MSCS-Emotion Regulation.

## Discussion

The objective of the current study was to assess two hypothesized multidimensional structures of a self-report version of the MSCS in a sample of young adults. First, the 7-first order and 1-second order model (i.e., Model 1) tested whether these 7 first-order domains all loaded onto the theorized construct of social competence. Second, the 7 first-order and 2 second-order model (i.e. Model 2) tested whether 3 of the first-order factors loaded onto the theorized construct of social responsiveness and the other 4 loaded onto the theorized construct of social understanding/emotion regulation. For each of these structural models, the SRMR and RMSEA indices suggested reasonably good fit. These results are consistent with previous findings [19].

Yager and Iarocci [19] theorized that the first-order domains of social motivation, empathic concern, and nonverbal sending skills might be indicative of individuals’ aptitudes for social responsiveness. In contrast, the first-order domains of social inferencing, social knowledge, verbal skills, and emotion regulation were theorized to represent social understanding and emotion regulation. These domains should be related as they each represent “the cognitive and behavioural skills needed to respond appropriately in social situations” [19]. Indeed, factor correlations between each of the first-order domains of the MSCS were comparable to that of Yager and Iarocci [19]. We found that the domains theorized to be related to the 2 higher order constructs were moderately to strongly correlated with one another. In contrast, we only found modest correlations between social motivation and verbal skills, and between emotion regulation and social motivation, empathic concern, social knowledge, and nonverbal sending skills. Emotion regulation scores were the least strongly correlated with the MSCS total score. The other domains all correlated quite strongly with the overall score.

A second aim was to determine whether forming scores at all three of the domain, subscale, and overall MSCS levels was justified for this sample. The results of our tests of the domain measurement models indicated adequate fit for all 7 domains of the MSCS. As such, forming sums of domain scores for each of these domains and interpreting these as indicators of single facets of social competence was justified for the current sample. In practice, researchers are encouraged to determine whether unidimensionality of each of these 7 domains holds for their own data and therefore whether non-weighted sums are justified.

Further validation of the MSCS in both clinical and non-clinical populations is warranted. In particular, item 41 in both parent-report and self-report versions of the MSCS needs further examination. Qualitative analyses of item descriptions might aid in identifying discrepancies in positive and negative factor loadings between different populations. In addition, item analyses to condense the items on the test will be useful. The current study presents a first step at validating the MSCS as a self-report and with diverse populations. However, to assess the utility of the MSCS to measure change over time requires longitudinal data.

Additionally, future research is needed to determine the convergent validity of the empathic concern and nonverbal sending skills domains, as the present study had no suitable scales to determine the convergent validity of these domains. In addition, some of the convergent validity indices presented in Table 5 were rather low small indicating questionable convergent validity of certain MSCS domains, although these small correlations may have been due to low reliability of the convergent measures (especially subscales of the AQ), or insufficient theoretical overlap with the MSCS domains. To provide a more complete validation study of the MSCS, future research will benefit from using convergent measures with stronger theoretical overlap, as well as the use of other methods besides self-report (e.g., other-report measures or “ecologically valid” measures of social competence based on real-world social behavior).

The MSCS is a first step to establishing some basic parameters of social competence that may be used as an index of a person’s social functioning. We do not mean for it to be a way to assess the “best” or only way to be socially competent as we acknowledge that social competence is a dynamic, context dependent construct that is socially constructed. There is a need for qualitative research to uncover the many ways that adults with ASD are resilient and competent in relationships that may be quite different from the TD way. However, the MSCS is based on the current literature on social development and, although limited, it can begin to point out the ways in which people (not just those with ASD) may be struggling to adapt to the mainstream social standards. This measure does not endorse mainstream social standards as the best or only way to be social. However, we acknowledge that people with ASD (and others who behave differently within a mainstream culture) are often in situations where they are interacting with people who abide by mainstream social standards and, as such, often struggle to adapt.

The MSCS has the potential to contribute to a variety of research applications in educational, clinical, and non-clinical settings. For example, MSCS profiles of social competence may prove useful in behavioural genetics research, in which a well specified phenotype or endophenotype is a more useful way of grouping participants than a clinical diagnosis [12]. Also, differentiating between “social responsiveness” and “social understanding/emotion regulation” components of social competence may be especially helpful in intervention studies.

## Supporting information

S1 File Multidimensional social competence scale. MSCS available for download.
(PDF)

S2 File Data used for analyses. Data file containing data used for all analyses in this study.
(SAV)
