# Supplementary material for: A psychometric evaluation of the Multidimensional Social Competence Scale (MSCS) for young adults
Source: PLoS One. 2018 Nov 2;13(11):e0206800. doi: 10.1371/journal.pone.0206800 (PMC6214554; doi:10.1371/journal.pone.0206800)
Supplement: S1 File — MSCS available for download. (PDF) [file pone.0206800.s001.pdf]

MSCS- Self Report

Jodi Yager, PhD and Grace Iarocci, PhD

Your Name (First, Middle, Last): ____________________________

Today's Date (Month, Day, Year): ___________________

Instructions: For each item, circle the number that best describes your behaviour over the **past six months**.

1 = Not True or Almost Never True
2 = Rarely True
3 = Sometimes True
4 = Often True
5 = Very True or Almost Always True

Many of the items may seem similar to one another, but your response on each one is very important. If you are unsure of an item, please put your best estimate.

1 = NOT TRUE OR ALMOST NEVER TRUE	2 = RARELY TRUE	3 = SOMETIMES TRUE	4 = OFTEN TRUE	5 = VERY TRUE OR ALMOST ALWAYS TRUE

**1.** I prefer to spend time alone (e.g., I am most content when left on my own).	1 2 3 4 5
**2.** I enjoy meeting new people.	1 2 3 4 5
**3.** I easily recognize unfriendly actions. For example, I know when someone is making fun of me in a mean-spirited way. Or, I recognize when a peer is pressuring me to do something I shouldn't or don't want to do.	1 2 3 4 5
**4.** I disagree with people without fighting or arguing.	1 2 3 4 5
**5.** I apologize after hurting someone (without being prompted or told to).	1 2 3 4 5
**6.** I talk "over" people in conversations (e.g., interrupt a lot, don't wait for others to finish speaking).	1 2 3 4 5
**7.** I shift conversations to my favourite topic or interest.	1 2 3 4 5
**8.** I talk about the same things over and over ("get stuck" on certain topics).	1 2 3 4 5
**9.** I am sensitive to the feelings and concerns of others.	1 2 3 4 5
**10.** I initiate friendly social "chit-chat" with people (e.g., ask about what's new with other person, talk about the weather or events). These are casual conversations that often have no specific purpose.	1 2 3 4 5
**11.** I appear visibly upset when I see people suffering (in real life or on tv/film).	1 2 3 4 5
**12.** I have trouble joining conversations appropriately (e.g., I may interrupt or "butt in" without waiting for a good time to join in; or, I may start talking about a topic of interest to me regardless of the ongoing conversation).	1 2 3 4 5
**13.** I misread social cues.	1 2 3 4 5
**14.** I stay in the "background" in group social situations (e.g., keep to myself, may not be noticed).	1 2 3 4 5

1 = NOT TRUE OR ALMOST NEVER TRUE	2 = RARELY TRUE	3 = SOMETIMES TRUE	4 = OFTEN TRUE	5 = VERY TRUE OR ALMOST ALWAYS TRUE

**15.** I am patient (e.g., when waiting).	1 2 3 4 5
**16.** I express concern for others when they are upset or distressed (e.g., may ask "are you alright?" or ask if they need anything).	1 2 3 4 5
**17.** I look people in the eye when talking to them.	1 2 3 4 5
**18.** I get frustrated easily.	1 2 3 4 5
**19.** I ask people questions about themselves or their lives (e.g., how they are, what they've been up to).	1 2 3 4 5
**20.** I do not offer to help people (unless asked or told to).	1 2 3 4 5
**21.** My emotional responses tend to be extreme (e.g., I might be extremely angry or frustrated in response to relatively small problems).	1 2 3 4 5
**22.** I avoid talking to people when possible (e.g., look, move, or walk away).	1 2 3 4 5
**23.** My facial expressions are easy to read.	1 2 3 4 5
**24.** I can tell when people are joking.	1 2 3 4 5
**25.** I stay calm when problems come up.	1 2 3 4 5
**26.** I know about the latest trends for my age (e.g., in clothes, music, tv shows/movies, music).	1 2 3 4 5
**27.** I am indifferent or "oblivious" to people who are upset (or in distress).	1 2 3 4 5
**28.** I pick up on subtle hints and indirect requests. For example, I would understand that when someone asks "Can you reach that book?", they are asking me to pass it to them. In other words, I can "read between the lines" when others are talking.	1 2 3 4 5
**29.** My smiles seem forced or awkward.	1 2 3 4 5
**30.** I give compliments to people.	1 2 3 4 5
**31.** I act appropriately for my age in public (e.g., restaurants, movie theatres, libraries, doctor's waiting rooms, etc).	1 2 3 4 5

**32.** I use eye contact to get other people's attention (e.g., to start a conversation, ask a question).	1 2 3 4 5
**33.** I change the volume of my voice depending on where I am (e.g., quiet at the library, movies but louder when outside or at a sporting event).	1 2 3 4 5
**34.** I show a range of facial expressions (e.g., embarrassed, guilty, surprised, disgusted, pleased).	1 2 3 4 5
**35.** I smile appropriately in social situations (e.g., if given a compliment, greeting someone, in response to someone smiling at me).	1 2 3 4 5
**36.** I act out when angry or upset (e.g., yell at, hit, or shove people).	1 2 3 4 5
**37.** I talk "at" people (e.g., almost like I am giving a lecture).	1 2 3 4 5
**38.** I go off track during conversations (e.g., I might change topics suddenly as if thinking aloud or reminded of something else; or, I might gradually get sidetracked or lose track of your original point).	1 2 3 4 5
**39.** I am concerned about people and their problems (e.g., talk to someone who is having a hard time).	1 2 3 4 5
**40.** I am naïve (believe whatever I am told).	1 2 3 4 5
**41.** I get over setbacks or disappointments quickly.	1 2 3 4 5
**42.** I need to be told or prompted to talk or interact with people.	1 2 3 4 5

1 = NOT TRUE OR ALMOST NEVER TRUE	2 = RARELY TRUE	3 = SOMETIMES TRUE	4 = OFTEN TRUE	5 = VERY TRUE OR ALMOST ALWAYS TRUE

**43.** I follow social "rules" around privacy (e.g., respect people's privacy when they are changing/ in the washroom; knock on closed doors instead of barging in).	1 2 3 4 5
**44.** I get very anxious.	1 2 3 4 5
**45.** I can see things from another person's perspective.	1 2 3 4 5
**46.** I have "meltdowns" (e.g., sudden outbursts, "blow ups" temper tantrums).	1 2 3 4 5
**47.** My expectations of friends reasonable. For example, I know that they have other friends or are not always available.	1 2 3 4 5
**48.** I offer comfort to people (e.g., to someone who is upset, not feeling well, hurt etc.). For instance, I may try to hug the person or provide a comforting object as a way of trying to make the other person feel better.	1 2 3 4 5
**49.** I use appropriate gestures when communicating with people (e.g., nodding/shaking head, waving goodbye, pointing at something interesting or far away, giving thumbs up, putting finger to lips for "be quiet", etc.).	1 2 3 4 5
**50.** I am good at taking turns in conversations (e.g., my conversations have age -appropriate levels of back-and-forth with each person getting a chance to talk; I respond appropriately to the other person's questions or statements).	1 2 3 4 5
**51.** My facial expressions seem "flat" (e.g., my face may be like a "blank slate" or seem overly serious).	1 2 3 4 5
**52.** I have trouble judging who is trustworthy (e.g., who to share secrets or personal information with).	1 2 3 4 5
**53.** I understand what makes a true friend.	1 2 3 4 5
**54.** I recognize when people are trying to take advantage of me.	1 2 3 4 5
**55.** I try to cheer people up (when they are down).	1 2 3 4 5
**56.** I give other people a chance to speak during conversations (e.g., pauses, asks them questions).	1 2 3 4 5

**57.** I seek out people to spend time with (e.g., friends, other people).	1 2 3 4 5
**58.** I understand the "social hierarchy" at school or work or in other settings (e.g., understand that teachers or supervisors are in a position of authority).	1 2 3 4 5
**59.** I have trouble predicting what other people will do or how they will react.	1 2 3 4 5
**60.** I get very upset if things are not done your way.	1 2 3 4 5
**61.** I dominate conversations so that it can be hard for others to "get a word in". For example, I might ramble on and on about a favourite topic of interest. I might also need reminders/prompting to let others speak.	1 2 3 4 5
**62.** I sound the same (have the same tone and intonation in his/her voice) regardless of how I am feeling. In other words, it is hard to tell what I am feeling based on the way my voice sounds.	1 2 3 4 5
**63.** I provide too much detail when talking about a topic (e.g., I might list a bunch of facts rather than expressing a main message or exchanging information).	1 2 3 4 5
**64.** I congratulate people when good things happen to them.	1 2 3 4 5
**65.** I initiate get-togethers with peers (e.g., call or email or text them to make plans).	1 2 3 4 5
**66.** I point at things when appropriate (e.g., to get another person to look at something far away).	1 2 3 4 5
**67.** I do not pick up on the subtleties of social interaction.	1 2 3 4 5

1 = NOT TRUE OR ALMOST NEVER TRUE	2 = RARELY ; TRUE	3 = SOMETIMES TRUE	4 = OFTEN TRUE	5 = VERY TRUE OR ALMOST ALWAYS TRUE

**68.** My emotions tend to be “all or nothing” (“all on” or “all off”).	1 2 3 4 5
**69.** I show little interest in people.	1 2 3 4 5
**70.** I speak with a flat, monotonous tone of voice.	1 2 3 4 5
**71.** I understand that it is important to have good personal hygiene (e.g., smelling and looking clean).	1 2 3 4 5
**72.** I change my behaviour to suit the situation. For example, I might be more polite/ formal around authority figures like teachers or supervisors but be more casual around peers. As another example, I might change my way of speaking depending on who you are talking to (e.g., talk more simply to a younger child).	1 2 3 4 5
**73.** I dress appropriately for my age and social situation (e.g., dress up for formal events, wear more casual clothes on weekends, wear clothes that are generally considered acceptable by peers my age).	1 2 3 4 5
**74.** I talk too much.	1 2 3 4 5
**75.** I hide my true feelings (when necessary) so that I don’t come across as rude (e.g., I might hide feelings of disappointment when given a gift that I do not like or when someone breaks something of mine by accident).	1 2 3 4 5
**76.** I introduce yourself to people (without being told to).	1 2 3 4 5
**77.** I understand when people are being sarcastic.	1 2 3 4 5

Self Report Feedback:

1. Were any items difficult to answer (provide item number) and explain why

____

____

____

____

2. Any comments or suggestions:

____

____

____

____

Copyright © 2014 Jodi Yager and Grace Iarocci. The MSCS Self Report (MSCS- SR) was developed by Jodi Yager and Grace Iarocci. All rights reserved, including translation. The profile for the MSCS- SR is designed to aid in diagnosis and treatment planning. No diagnosis or treatment decisions should be made solely on the basis of this report without confirming information from independent sources.
